# Thalamic mediodorsal nucleus and its participation in spatial working memory processes: comparison with the prefrontal cortex

**DOI:** 10.3389/fnsys.2013.00036

**Published:** 2013-07-31

**Authors:** Shintaro Funahashi

**Affiliations:** Kokoro Research Center, Kyoto UniversityKyoto, Japan

**Keywords:** thalamic mediodorsal nucleus, prefrontal cortex, spatial working memory, delayed-response, retrospective information, prospective information

## Abstract

Working memory is a dynamic neural system that includes processes for temporarily maintaining and processing information. Working memory plays a significant role in a variety of cognitive functions, such as thinking, reasoning, decision-making, and language comprehension. Although the prefrontal cortex (PFC) is known to play an important role in working memory, several lines of evidence indicate that the thalamic mediodorsal nucleus (MD) also participates in this process. While monkeys perform spatial working memory tasks, MD neurons exhibit directionally selective delay-period activity, which is considered to be a neural correlate for the temporary maintenance of information in PFC neurons. Studies have also shown that, while most MD neurons maintain prospective motor information, some maintain retrospective sensory information. Thus, the MD plays a greater role in prospective motor aspects of working memory processes than the PFC, which participates more in retrospective aspects. For the performance of spatial working memory tasks, the information provided by a sensory cue needs to be transformed into motor information to give an appropriate response. A population vector analysis using neural activities revealed that, although the transformation of sensory-to-motor information occurred during the delay period in both the PFC and the MD, PFC activities maintained sensory information until the late phase of the delay period, while MD activities initially represented sensory information but then started to represent motor information in the earlier phase of the delay period. These results indicate that long-range neural interactions supported by reciprocal connections between the MD and the PFC could play an important role in the transformation of maintained information in working memory processes.

## Introduction

Working memory is a dynamic neural system that includes neural processes for temporarily maintaining and processing information. Working memory is a fundamental neural component for a variety of cognitive functions, such as thinking, reasoning, decision-making, and language comprehension (Baddeley and Hitch, 1974; Baddeley, 2000). Therefore, working memory is an important concept for understanding the neural mechanisms of higher cognitive functions.

Working memory is an important concept for understanding the roles of the dorsolateral prefrontal cortex (DLPFC) in a variety of cognitive functions (Goldman-Rakic, 1987; Funahashi and Kubota, 1994; Funahashi, 2001; Funahashi and Takeda, 2002; Fuster, 2008). Brain imaging studies in human subjects have revealed that the DLPFC is activated whenever subjects perform behavioral tasks that require working memory (Stuss and Knight, 2012). Neuropsychological studies have also revealed that damage to the DLPFC impairs the performance of working memory tasks in human subjects (Stuss and Levine, 2002; Stuss et al., 2002). In animal studies, lesion of the DLPFC impaired performance in behavioral tasks that included an imposed delay period between cue presentation and response generation (e.g., delayed-response, delayed alternation, delayed matching-to-sample task) (Fuster, 2008). In neurophysiological studies, tonic sustained excitatory activity during the delay period (delay-period activity) has been observed in DLPFC neurons while monkeys performed behavioral tasks with a delay (Funahashi et al., 1989). These findings support the notion that the DLPFC plays an essential role in working memory processes.

However, the DLPFC is not the only brain area that participates in working memory processes. Neurophysiological studies using monkeys have shown that neurons in the parietal cortex (Gnadt and Andersen, 1988; Chafee and Goldman-Rakic, 1998), the temporal cortex (Fuster and Jervey, 1982; Miller et al., 1991, 1993), and the basal ganglia (Hikosaka and Sakamoto, 1986; Hikosaka et al., 1989) exhibit tonic sustained excitatory activity during the delay period. All of these brain areas have anatomical connections to the DLPFC (Selemon and Goldman-Rakic, 1988; Fuster, 2008). Therefore, these brain areas also play important roles in working memory and might construct neural circuitries for working memory with the DLPFC.

The mediodorsal nucleus (MD) of the thalamus MD also has strong reciprocal connections with the prefrontal cortex (PFC) (Kievit and Kuypers, 1977; Goldman-Rakic and Porrino, 1985; Giguere and Goldman-Rakic, 1988; Ray and Price, 1993). Since the thalamus is the major relay structure that provides information to the cerebral cortex, it has been described as a gateway to the cortex (Sherman and Guillery, 2006). However, recent studies have indicated that, not only is the thalamus the gateway to the cerebral cortex, it also significantly contributes to cognitive functions. In fact, several lines of physiological evidence indicate that the MD participates in working memory processes in monkey experiments (Fuster and Alexander, 1971, 1973; Kubota et al., 1972; Tanibuchi and Goldman-Rakic, 2003). The experiment using rats also indicates that the MD participates in cognitive functions, such as prefrontal-dependent cognitive behaviors (Parnaudeau et al., 2013).

In this article, I will focus on the participation of the thalamus in cognitive functions. To demonstrate the importance of the thalamus in cognitive functions, I focus on working memory as an example of cognitive functions and the MD as a thalamic nucleus, and explain how the MD contributes to working memory processes. To explain the contribution of the MD to working memory, related findings obtained in prefrontal studies are helpful. Therefore, I will first explain findings regarding the neural mechanisms of working memory in the PFC, then explain the neural mechanisms of working memory in the MD, and finally consider the functions of the MD in a model of working memory.

## Working memory processes in the dorsolateral prefrontal cortex

### Mechanisms of spatial working memory in the dorsolateral prefrontal cortex

Since Goldman-Rakic (1987) proposed that working memory is an important concept for understanding the functions of the DLPFC in both humans and animals, the importance of the DLPFC in working memory has been demonstrated in a variety of experiments including lesion studies (see reviews by Goldman-Rakic, 1987; Petrides, 1994; Fuster, 2008), brain imaging studies using human subjects (see Stuss and Knight, 2012), and neurophysiological studies using non-human primates (see reviews by Funahashi and Kubota, 1994; Goldman-Rakic, 1998; Funahashi and Takeda, 2002; Fuster, 2008). Neurophysiological studies have shown that many neurons in the DLPFC exhibit tonic sustained activation during the delay period (delay-period activity) while monkeys performed spatial working memory tasks (Fuster, 1973; Niki, 1974; Niki and Watanabe, 1976; Kojima and Goldman-Rakic, 1984; Joseph and Barone, 1987; Funahashi et al., 1989; Hasegawa et al., 1998).Delay-period activity has been shown to have several important features regarding the neural mechanisms of working memory. First, the duration of delay-period activity can be prolonged or shortened depending on the length of the delay period (Fuster, 1973; Kojima and Goldman-Rakic, 1984; Funahashi et al., 1989). Second, this activity is observed only when monkeys perform correct behavioral responses (Fuster and Alexander, 1973; Funahashi et al., 1989). When the monkey made an error, delay-period activity was either truncated or not observed in that trial. Third, a great majority of delay-period activity exhibits a directional or positional preference (Funahashi et al., 1989), such that delay-period activity was observed only when a visual cue was presented at a particular area in the visual field. Many DLPFC neurons exhibited directional delay-period activity, and the preferred direction of this activity differed from neuron to neuron. Therefore, it has been proposed that neurons that exhibit directional delay-period activity have mnemonic receptive fields (memory fields) in the visual field (Funahashi et al., 1989; Rainer et al., 1998), analogous to visual receptive fields. Fourth, with the use of a delayed pro- and anti-saccade task, it has been shown that the great majority (about 70%) of delay-period activity represented information regarding the position of the visual cue (retrospective information), whereas the minority (about 30%) represented information regarding the direction of the saccade (prospective information) (Funahashi et al., 1993). Takeda and Funahashi (2002) used a conventional oculomotor delayed-response (ODR) task and a modified version (R-ODR task). In the ODR task, monkeys were required to make a saccade toward the direction of the visual cue after the delay, whereas in the R-ODR task, monkeys were required to make a saccade 90° clockwise from the direction of the visual cue. They compared the best directions of delay-period activity between these two task conditions. If the best directions of delay-period activity were the same in these two conditions, delay-period activity would encode the direction of the visual cue, since the best direction was depicted using the direction of the visual cue in their experiments. However, if the best directions of delay-period activity showed a 90° difference, delay-period activity would encode the direction of the saccade. They found that a great majority of delay-period activity (86%) encoded the direction of the visual cue, while a minority (13%) encoded the direction of the saccade. Similarly, Niki and Watanabe (1976) used a manual delayed-response task and a conditional position task, and reported that 70% and 30% of DLPFC neurons represented the spatial position of the visual cue and the direction of the response, respectively. Thus, delay-period activity represents either retrospective or prospective information, although most delay-period activity represents retrospective information in the DLPFC. Based on these observations, delay-period activity has been considered to be a neural correlate of temporary information-storage processes (Goldman-Rakic, 1987; Funahashi and Kubota, 1994; Miller, 2000; Funahashi, 2001; Funahashi and Takeda, 2002; Fuster, 2008).

Although the above observations were obtained using spatial working memory tasks, experiments with non-spatial working memory tasks (e.g., delayed matching-to-sample tasks and delayed conditional tasks) have also revealed that delay-period activity represents the active retention of non-spatial information, such as object shapes, patterns, or colors (Sakagami and Niki, 1994; Miller et al., 1996; Rao et al., 1997; Asaad et al., 1998; Rainer et al., 1999; Freedman et al., 2002). In addition, Romo et al. (1999) showed that differences in somatosensory information (e.g., frequency of mechanical vibrations) were encoded by the difference in the magnitude of delay-period activity in DLPFC neurons. Further, delay-period activity has been shown to encode reward information and to be affected by the preference for the reward (Watanabe, 1996; Hikosaka and Watanabe, 2000; Kobayashi et al., 2002; Wallis and Miller, 2003). These results indicate that delay-period activity can represent not only spatial information but also non-spatial information, and confirm that delay-period activity observed in the DLPFC is a neural correlate of the mechanism for temporarily maintaining a variety of information (Funahashi and Kubota, 1994; Goldman-Rakic, 1998; Miller, 2000; Funahashi, 2001; Funahashi and Takeda, 2002; Fuster, 2008). Neurons with various task-related activities and neurons that exhibited various spatial and non-spatial features in task-related activity were distributed widely throughout the DLPFC with substantial overlap (Carlson et al., 1997; Quintana and Fuster, 1999; Rainer et al., 1999; Sakagami and Tsutsui, 1999). In addition, several neurons exhibited delay-period activity in both spatial and non-spatial working memory tasks (Rao et al., 1997). Therefore, neurons in the DLPFC can maintain various types of information as tonic sustained delay-period activity. Since each neuron exhibits a different preference for information and maintains it as delay-period activity, different information can be encoded by different groups of DLPFC neurons.

### Information processing in the dorsolateral prefrontal cortex during spatial working memory processes

Information processing in working memory can be considered as altering or transforming temporarily stored information in an appropriate way for accomplishing a particular purpose. Therefore, information processing could be achieved by dynamical and flexible functional interactions among mechanisms for temporarily storing information. Neurophysiological studies have provided evidence for the alteration or transformation of information by functional interactions among DLPFC neurons. Several studies have shown that the information represented by prefrontal activity changes as the task progresses. For example, in a paired association task with a delay, prefrontal activity represented the characteristics of the sample stimuli (sensory-related retrospective coding) in the early phase of the delay period, but began to represent the characteristics of anticipated targets (prospective coding) toward the end of the delay period (Rainer et al., 1999). Similarly, in a spatial delayed matching-to-sample task, spatial information was broadly tuned by delay-period activity in the early phase of the delay period. However, the proportion of neurons that exhibited sharper spatial tuning and high spatial discriminability increased in the later phase of the delay period (Sawaguchi and Yamane, 1999). Further, Asaad et al. (1998) showed that neural activity conveyed the direction of an impending eye movement progressively earlier along successive trials while monkeys performed arbitrary cue-response association tasks. Quintana and Fuster (1999) observed neurons attuned to the cue color and neurons attuned to the response directions while monkeys performed working memory tasks using color cues. They found that the discharge of neurons attuned to the cue color gradually diminished during the delay period, whereas the discharge of neurons attuned to the response directions gradually increased. All of these results indicate that the alteration of the neuron’s discharge rate as the delay period progresses reflects the alteration of the information represented by the neuron. Thus, the temporal change in firing patterns observed in a population of neurons could reflect the progress of information processing during the delay period.

Takeda and Funahashi (2004) used a population vector analysis and demonstrated a temporal change in the preferred direction encoded by a population of DLPFC neurons as the delay period progressed in two ODR tasks (ODR and R-ODR tasks). In the ODR task, the monkey was required to make a saccade to the direction where the visual cue was presented, whereas in the R-ODR task the monkey was required to make a saccade 90° clockwise from the direction where the visual cue was presented. Takeda and Funahashi (2002) indicated two groups of DLPFC neurons with delay-period activity that encoded either the direction of the visual cue or the direction of the saccade, respectively. In the ODR task, since the direction of the visual cue is the same as the direction of the saccade, the preferred direction encoded by a population of DLPFC neurons would be maintained throughout the delay period. However, in the R-ODR task, the direction of the saccade is 90° clockwise from the direction of the visual cue. Therefore, the preferred direction encoded by a population of DLPFC neurons would change from the direction of the visual cue to the direction of the saccade during the delay period.

**Figure 1:**
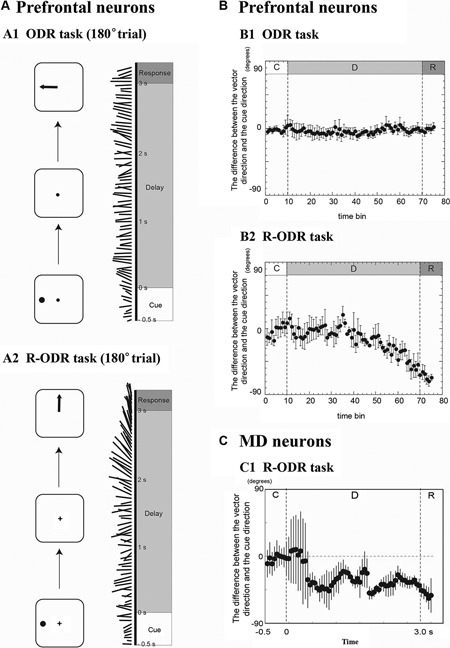
**Temporal changes in the directions of population vectors in prefrontal neurons (A and B) and MD neurons (C)**. **(A1)** Temporal changes in the directions of population vectors during the 180° trial of the ODR task. Most population vectors were directed toward the 180° direction. **(A2)** Temporal changes in the directions of population vectors during the 180° trial of the R-ODR task. The direction of the population vector gradually rotated from the 180° direction to the 90° direction during the delay period. **(B1)** The difference between the vector direction and the cue direction during the ODR trial. The population vector was directed toward the cue direction during the delay period. **(B2)** The difference between the vector direction and the cue direction during the R-ODR trial. The direction of the population vector gradually rotated from the cue direction to the saccade direction during the delay period. The timing of the change was start at 1.5 s after the start of the delay period (adapted from Takeda and Funahashi (2004)). **(C1)** Temporal changes in the differences between the directions of population vectors and the direction of the visual cue in the R-ODR task for MD neurons. The direction of the population vector rotated from the cue direction to the saccade direction during the delay period, similar as prefrontal neurons. However, the timing of the change was start at 0.5 s after the start of the delay period (Adapted from Watanabe et al. (2009)).

Figure 1-A1 shows population vectors calculated from a population of DLPFC activities in the 180° trial of the ODR task. Since the direction of the visual cue and the direction of the saccade were the same in the ODR task, population vectors were mostly directed toward the 180° direction. Figure 1-B1 shows temporal changes in the directions of population vectors across all four conditions and confirms that the directions of the population vectors are the same as the direction of the visual cues and are maintained during the delay period. Figure 1-A2 shows population vectors calculated for a population of DLPFC activities in the 180° trial of the R-ODR task. In this trial, the visual cue was presented at the 180° direction but the correct saccade was in the 90° direction. Population vectors were directed toward the 180° direction at the beginning of the delay period. However, the population vectors began to rotate in the middle of the delay period, continued to rotate slowly from the 180° direction to the 90° direction during the late half of the delay period, and were eventually directed toward the 90° direction at the response period (see Figure 1-B2). These results indicate that the information represented by a population of DLPFC activities changes from sensory information to motor information during the delay period, since the initial information is provided by sensory cues and must be transformed into motor information in these behavioral tasks. A population vector analysis can visualize the process for this transformation of information. Fuster (2008) stated that the delay period is the period for cross-temporal bridging when the transformation of sensory-to-motor information occurs, which is a dynamic process for the internal transfer of information as well as a process of cross-temporal matching. The present result indicates that the DLPFC plays a significant role in mediating the cross-temporal contingency. This result also indicates that DLPFC neurons contribute significantly to dynamic neural processes for internal information transfer.

### Importance of functional interaction in working memory in the dorsolateral prefrontal cortex

When we consider the neural mechanism of information processes in working memory, the essential components must be dynamic and flexible functional interactions among neurons that exhibit different task-related activities, different types of activity, and different directional selectivity. In the DLPFC, Wilson et al. (1994) showed that the types of responses (excitatory or inhibitory) of pyramidal neurons were often opposite those of non-pyramidal neurons (e.g., when pyramidal neurons exhibited an excitatory response, non-pyramidal neurons often exhibited an inhibitory response). They also showed that the timing of excitatory and inhibitory responses appears to be anti-phased between pyramidal and non-pyramidal neurons. These results indicate the presence of functional interactions between pyramidal and non-pyramidal neurons in the DLPFC. Further, Rao et al. (1999) found inhibitory interactions between a pyramidal neuron and an adjacent non-pyramidal interneuron by cross-correlation analyses of neuronal firing in the DLPFC. Funahashi and Inoue (2000) also examined functional interactions between task-related DLPFC neurons by cross-correlation analyses. When both neurons of examined pairs exhibited delay-period activity, these neurons tended to have excitatory interactions and showed similar directional preferences. An examination of the temporal change in the strength of correlated firings revealed that functional interactions between task-related neurons with different directional preferences increased as the trial progressed. These observations suggest that the information represented by a population of neurons that exhibits directional delay-period activity gradually transforms into other types of information by these functional interactions, as indicated by a population vector analysis with a population of DLPFC activities.

The magnitude of activity of each neuron changes depending on the trial conditions, the temporal context of the trial, and the trial events. Therefore, the strength of functional interactions could change depending on the trial conditions or the temporal context of the trial. In fact, the strength of the cross-correlation calculated from the activities of two neurons recorded simultaneously changed dynamically depending on the cue conditions. Thus, dynamic and flexible changes in functional interactions among neurons are important components of neural mechanisms of information processing.

## Working memory processes in the thalamic mediodorsal nucleus

### The mediodorsal nucleus and working memory

The thalamus consists of several thalamic nuclei, each of which has reciprocal connections with specific regions of the cerebral cortex. The MD is a major thalamic nucleus and is located at the midline of the thalamus. An important feature of the MD is that it has strong reciprocal connections, mainly to the PFC. Therefore, the MD could also play significant roles in a variety of higher cognitive functions in which the PFC participates, including working memory.

Animal studies have shown that the MD participates in working memory processes. Lesion of the monkey MD has been shown to impair performance in working memory tasks. For example, Isseroff et al. (1982) found that lesions in the monkey MD were associated with impairment in a spatial delayed alternation task and a delayed-response task, while there was no impairment in an object reversal task or a visual pattern discrimination task. Since spatial working memory capacity is required for the former two tasks, but not for the latter two tasks, they concluded that lesion of the MD impaired spatial working memory capacity. Lesion of the monkey MD also impaired performance in non-spatial working memory tasks including a delayed matching-to-sample task (Aggleton and Mishkin, 1983a,b; Parker et al., 1997) and a delayed non-matching-to-sample task (Zola-Morgan and Squire, 1985). Alexander and Fuster (1973) examined functional interactions between the DLPFC and the MD by cooling of the DLPFC in monkeys and found that the activities of most (63%) MD neurons were affected by cooling of the DLPFC. The cooling effects observed in MD neurons included the attenuation of delay-period activity, shortening of the duration of delay-period activity, and the inhibition of delay-period activity.

The human MD has also been shown to participate in working memory. Damage to the medial thalamus including the MD often produces syndromes similar to “prefrontal syndromes” in humans (Daum and Ackermann, 1994; Van der Werf et al., 2000, 2003). The impairment of executive function is a major symptom of “prefrontal syndromes” (see Stuss and Benson, 1986). Working memory is a fundamental neural process of executive function (Funahashi, 2001). Therefore, the impairment of executive function due to damage to the medial thalamus could be caused by the impairment of working memory. For example, Van der Werf et al. (2003) used four neuropsychological tests (Wisconsin card sorting test, Tower of London test, verbal category fluency test, and Stroop test) to assess executive function in 22 patients with thalamic infarction. They found that patients with damage in the MD exhibited impaired performance in all of these neuropsychological tests. Since all of these tests require working memory capacity, this result indicates that the human MD also participates in working memory. Zoppelt et al. (2003) also examined the relation between dysfunction of executive ability and the anatomical locus of the damaged area in the thalamus. For patients with thalamic infarction, the anatomical locus of the damaged area was identified by MRI. Among five patients with damage in the MD, two had damage predominantly in the medial MD and three had damage predominantly in the lateral MD. The capacity of executive function was assessed by the Stroop test, a verbal fluency task, and digit span tests (forward and backward reproduction). They found that patients with damage in the lateral MD exhibited more severe impairment in digit span tests with backward reproduction and in the phonemic condition of the verbal fluency test, whereas patients with damage in the medial MD did not exhibit impairment in these tests. These results indicate that the lateral MD is important for executive function. They also showed that, although patients with MD damage showed impaired memory processes such as recollection and familiarity, these memory impairments were more apparent when the damaged area included the medial MD. Anatomical studies have shown that the lateral MD has anatomical connections mainly with the DLPFC, whereas the medial MD has anatomical connections mainly with the orbitofrontal cortex (Kievit and Kuypers, 1977; Goldman-Rakic and Porrino, 1985; Giguere and Goldman-Rakic, 1988). Thus, the participation of the MD in cognitive functions seems to depend on its anatomical relations to the PFC (Rovo et al., 2012). Since the lateral MD has anatomical connections with the DLPFC and since the DLPFC participates in working memory processes, the lateral MD could play an important role in executive functions and working memory.

Functional brain imaging studies have also demonstrated that the human MD participates in working memory processes. Activation of the human MD has been observed while subjects performed working memory tasks, such as delayed matching-to-sample tasks and delayed non-matching-to-sample tasks (Elliott and Dolan, 1999; de Zubicaray et al., 2001). In addition to the temporary maintenance of information in working memory, Monchi et al. (2001) showed that the MD participated in other aspects of information processing. They asked human subjects to perform the Wisconsin card sorting test and control tasks and examined thalamic activation using fMRI. They found that the MD was activated when the subjects received negative feedback. In the Wisconsin card sorting test, negative feedback signals the subject to shift the category for selection from that used in the preceding trial to a new one. Thus, the MD participates not only in the temporary maintenance of information but also in information processing, such as in the replacement of the content of working memory (current category) with new information (new category).

### Neural activity related to working memory in the mediodorsal nucleus

**Figure 2:**
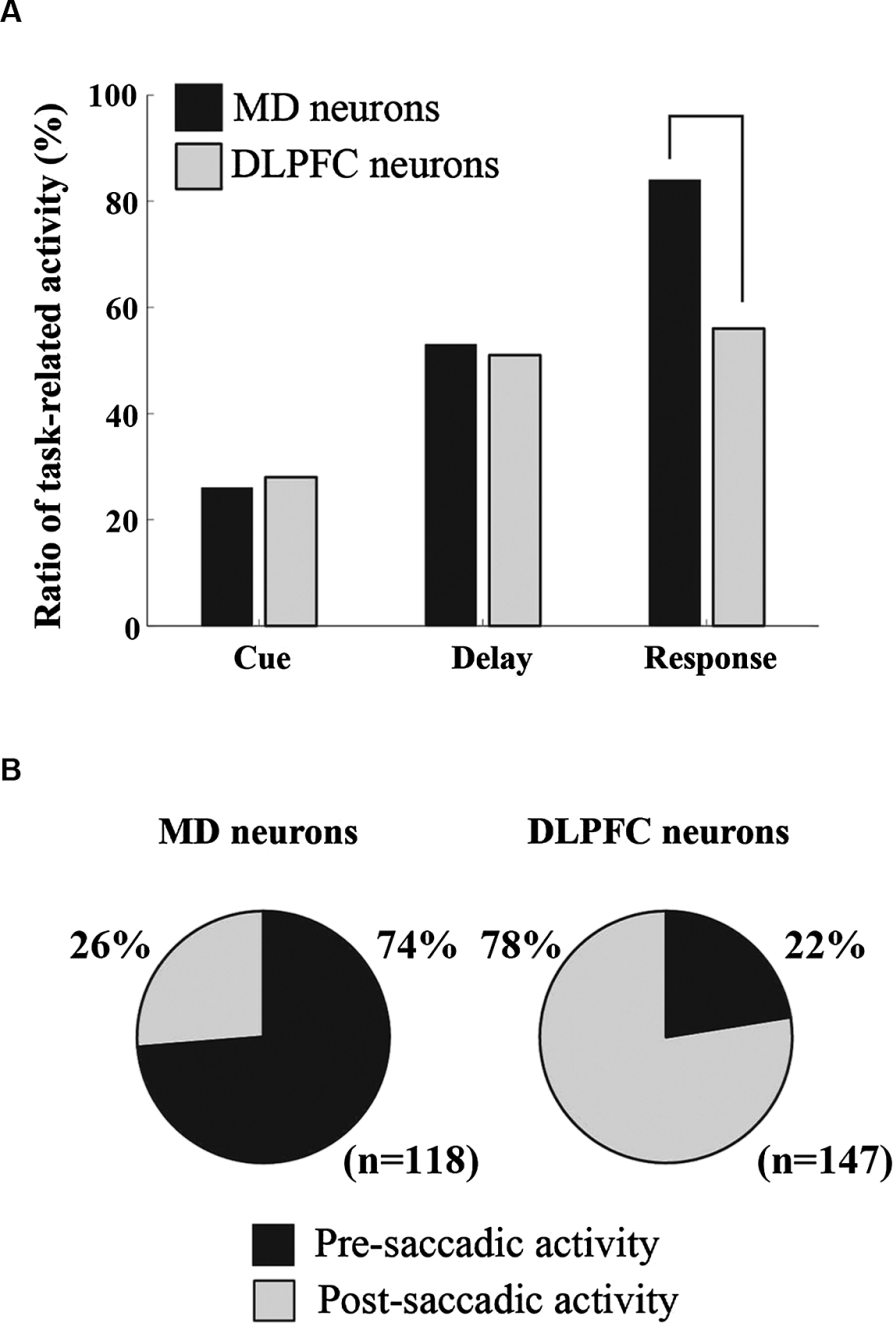
**Comparison of the characteristics of task-related activity between MD neurons and DLPFC neurons.** **(A)** A comparison of the proportion of task-related activity between the MD and DLPFC. **(B)** A comparison of the proportion of pre- and post-saccadic activity between the MD and DLPFC. The data regarding DLPFC neurons and MD neurons are based on data obtained by Funahashi et al. (1989, 1990, 1991) and Watanabe and Funahashi (2004a), respectively.

Neurophysiological studies with monkeys have demonstrated neural activity that was related to working memory, such as delay-period activity, in the MD. Fuster and Alexander (1971, 1973) first showed that about half of the recorded MD neurons exhibited sustained excitatory activity during the delay period (delay-period activity) while monkeys performed a delayed-response task. Watanabe and Funahashi (2004a) analyzed the characteristics of task-related activity of MD neurons while monkeys performed an ODR task. Since the same ODR task had been used to examine the neural mechanisms of working memory processes in the DLPFC (Funahashi et al., 1989, 1990, 1991, 1993; Takeda and Funahashi, 2002), it would be worthwhile to compare the characteristics of neural activities recorded using the same task in the MD and the DLPFC. Among recorded MD neurons, 26%, 53%, and 84% exhibited cue-, delay-, and response-period activity, respectively. Comparison of these values between the MD and the DLPFC indicated that more neurons exhibited response-period activity in the MD than in the DLPFC (Figure 2A). Among MD neurons with response-period activity, 74% showed pre-saccadic activity, while the remaining 26% showed post-saccadic activity. In contrast, a great majority (78%) of response-period activity was post-saccadic in the DLPFC (Figure 2B). Thus, the percentage of neurons with pre- or post-saccadic activity is an important difference in the functional characteristics of the MD and the DLPFC.

Task-related activity observed in the ODR task showed directional selectivity in MD neurons. For example, all cue-period activity, 76% of delay-period activity, and 64% of response-period activity showed directional selectivity. A similar directional selectivity of MD neurons was reported by Tanibuchi and Goldman-Rakic (2003). Among response-period activities, 78% of pre-saccadic activity and 26% of post-saccadic activity was directionally selective (Watanabe and Funahashi, 2004a). The proportion of directionally selective task-related activity was similar in the MD and the DLPFC (Funahashi et al., 1989, 1990, 1991; Takeda and Funahashi, 2002). Most task-related activity showed directional selectivity in both MD and DLPFC neurons.

Since most task-related activity exhibited directional selectivity, we could determine a preferred direction for each task-related activity based on a tuning curve constructed by recorded neural activities. In MD neurons, statistically significant contralateral bias in preferred directions was present in both cue-period activity and pre-saccadic activity, while significant contralateral bias was not observed in delay-period activity and most post-saccadic activity exhibited omni-directional selectivity (Watanabe and Funahashi, 2004a). In contrast, in DLPFC neurons, a statistically significant contralateral bias of preferred directions was observed in cue-period activity, delay-period activity, and pre-saccadic activity, while significant contralateral bias was not observed in post-saccadic activity (Funahashi et al., 1989, 1990, 1991; Takeda and Funahashi, 2002).

These results indicate that, while monkeys performed the ODR task, similar types of task-related activity were observed in similar proportions in the MD and the DLPFC. Directional delay-period activity was observed in the MD with similar characteristics and a similar proportion as in the DLPFC. Therefore, these findings strongly support the idea that the MD participates in spatial working memory processes. However, the MD and the DLPFC also show some differences, especially in response-period activity. Response-period activity was more frequently observed in the MD (84%) than in the DLPFC (56%), and the proportion of pre-saccadic activity in the MD (74%) was greater than that in the DLPFC (22%). Thus, although the MD and the DLPFC both participate in working memory processes, the MD contributes more to prospective aspects of working memory processes, such as motor or response preparation, compared to the DLPFC, which contributes more to retrospective aspects, such as sensory processes.

### Representation of information in the activity of mediodorsal nucleus neurons

Somewhat different contributions to working memory processes in the MD and the DLPFC are also observed when we examine the type of information that is encoded by delay-period activity. Watanabe and Funahashi (2004b) used the same ODR tasks as Takeda and Funahashi (2002) and examined the type of information encoded by the delay-period activity of MD neurons. They found that 56% of delay-period activity encoded the direction of the visual cue, while 41% encoded the direction of the saccade. Thus, more delay-period activity encoded the direction of the saccade in MD neurons than in DLPFC neurons. Together with the finding that more MD neurons exhibited response-period activity and most response-period activity showed pre-saccadic activity in the MD, these results support the idea that the MD participates more in motor aspects of working memory processes than the DLPFC and might provide impending motor information (prospective information) to the DLPFC.

### Population vector analysis using mediodorsal nucleus neural activities

A population vector analysis was applied to MD activities to visualize information processes in the MD while monkeys performed spatial working memory tasks (ODR and R-ODR tasks) (Watanabe et al., 2009). After the authors confirmed that population vectors constructed by a population of cue- and response-period activities correctly represented information regarding the directions of the visual cue and the saccade, respectively, they calculated population vectors of MD activities during a 250 ms window which slid in 50 ms steps from the onset of the visual cue until 500 ms after the initiation of the response period (Figure 1-C1). In the ODR task, the directions of population vectors were maintained mostly toward the direction of the visual cue throughout the entire delay period. In the R-ODR task, the direction of the population vector was initially in the direction of the visual cue, then began to rotate toward the direction of the saccade in the early phase of the delay period, and gradually pointed toward the direction of the saccade as the trial progressed. These results indicate that the transformation from visual information to saccade information occurs during the delay period in the MD. In addition, comparison of the temporal change in the directions of population vectors of DLPFC neurons and MD neurons revealed that the rotation of the population vector started earlier in the MD than in the DLPFC (Figure 1). In addition, as we considered previously, more delay-period activity encoded the direction of the saccade and more response-period activity exhibited pre-saccadic activity in the MD compared with the DLPFC. These results indicate that the MD might be the major brain area that provides information regarding impending motor information to the DLPFC.

### The mediodorsal nucleus and motor aspects of information processing

Although the MD participates in cognitive functions such as working memory, other results also support the idea that the MD contributes more to motor aspects rather than sensory aspects. For example, Sommer and Wurtz (2004) examined neural signals conveyed through an ascending pathway from the superior colliculus (SC) to the frontal eye field (FEF) via the MD. They used antidromic and orthodromic responses generated by electrical stimulation of the FEF to identify relay neurons in the MD. They examined the nature of the information that was transferred from the SC to the FEF while monkeys performed delayed-saccade tasks. They found that, although the SC sent visual as well as saccade signals to the FEF via the MD, pre-saccadic activity was prominent in MD relay neurons. Based on these and other results, they hypothesized that a major signal conveyed by the ascending pathway to the FEF is the corollary discharge that represents information regarding the direction and amplitude of an impending saccade (Sommer and Wurtz, 2002, 2004). In addition to the SC, the basal ganglia also project to the thalamus, including the MD, and provide information regarding saccades (Hikosaka et al., 2000). For example, an anatomical study by Ilinsky et al. (1985) showed that the substantia nigra has wide projections to the whole area of the MD. It has been known that neurons in the substantia nigra exhibit saccade-related activity (Hikosaka and Wurtz, 1983). High frequency tonic activity observed in the substantia nigra has inhibitory effect to thalamic neurons and this tonic activity temporarily suppresses thalamic activity in relation to the saccade performance. Therefore, thalamic neurons are disinhibited during saccade performance. Thus, activity of thalamic neurons is controlled by movement-related inputs from the basal ganglia.

Based on a comparison of the best directions of delay-period activity in the ODR and R-ODR tasks, most MD neurons encoded impending saccade information in delay-period activity. A population vector analysis revealed that impending saccade information was generated in the earlier phase of the delay period in the MD, while the same information was generated in the later phase of the delay period in the DLPFC. More pre-saccadic activity was observed in the MD than in the DLPFC. In addition, the MD received corollary discharge that represented information regarding the direction and amplitude of an impending saccade from the SC and sent this signal to the PFC. These results indicate that the MD is one of the brain structures that provide forthcoming motor information (prospective information) to the DLPFC. While we do not yet fully understand how prospective motor information is generated and which brain structures provide prospective motor information to the MD, the SC is one of these structures. Retrospective sensory information maintained in the DLPFC may also play a role to produce prospective motor information in the MD. Further studies are needed to understand how prospective motor information is generated and which brain areas participate in this process.

## Contributions of the mediodorsal nucleus to spatialworking memory processes in the DLPFC

We previously proposed neural components to explain spatial working memory processes based on our findings obtained from neurophysiological studies in the DLPFC (Funahashi, 2001). We hypothesized the presence of four basic neural components to execute working memory. These include a neural process for selecting appropriate information (selection process), a neural process for temporarily storing information (temporary storage process), a neural process for providing stored information to other neural systems (output process), and a neural process for appropriately processing the information (operation process). Working memory is defined as a system that includes both the temporary maintenance of and processing of information. Therefore, the temporary storage and operation processes are considered to be essential neural components of working memory. In addition to these neural components, the neural process for temporarily storing information can receive various kinds of information, including sensory, motor, motivational, emotional, cognitive, and perhaps somatic information. However, necessary and important information for executing the current task or achieving the current goal needs to be selected from among these varieties of information. Therefore, the neural process for working memory must include a neural process for selecting appropriate information from a variety of sources. In addition, stored and processed information should be used to perform the current task. For this purpose, the neural process for working memory must have a neural process to provide stored and processed information to other neural systems. Thus, when we consider a physiologically plausible model of working memory, the model should include at least these four neural processes.

We proposed four neural processes to explain how working memory function is executed in the DLPFC. However, it is hard to imagine how information processing could be a distinct neural component. Therefore, we hypothesize that information processing can be explained as a variety of functional interactions among temporary storage processes. The presence of various functional interactions among DLPFC neurons has been shown by neurophysiological studies. For example, excitatory as well as inhibitory interactions have been observed among task-related DLPFC neurons by a cross-correlation analysis of simultaneously recorded pairs of single-neuron activities (Funahashi and Inoue, 2000; Constantinidis et al., 2001). Dynamic and flexible interactions among neurons that depend on the progress of the trial have also been observed in the DLPFC by an analysis that used joint peri-stimulus time histograms (Vaadia et al., 1995; Funahashi, 2001; Tsujimoto et al., 2008). Thus, dynamic and flexible interactions among neural processes, especially among temporary storage processes, could play an essential role in information processing in working memory.

**Figure 3:**
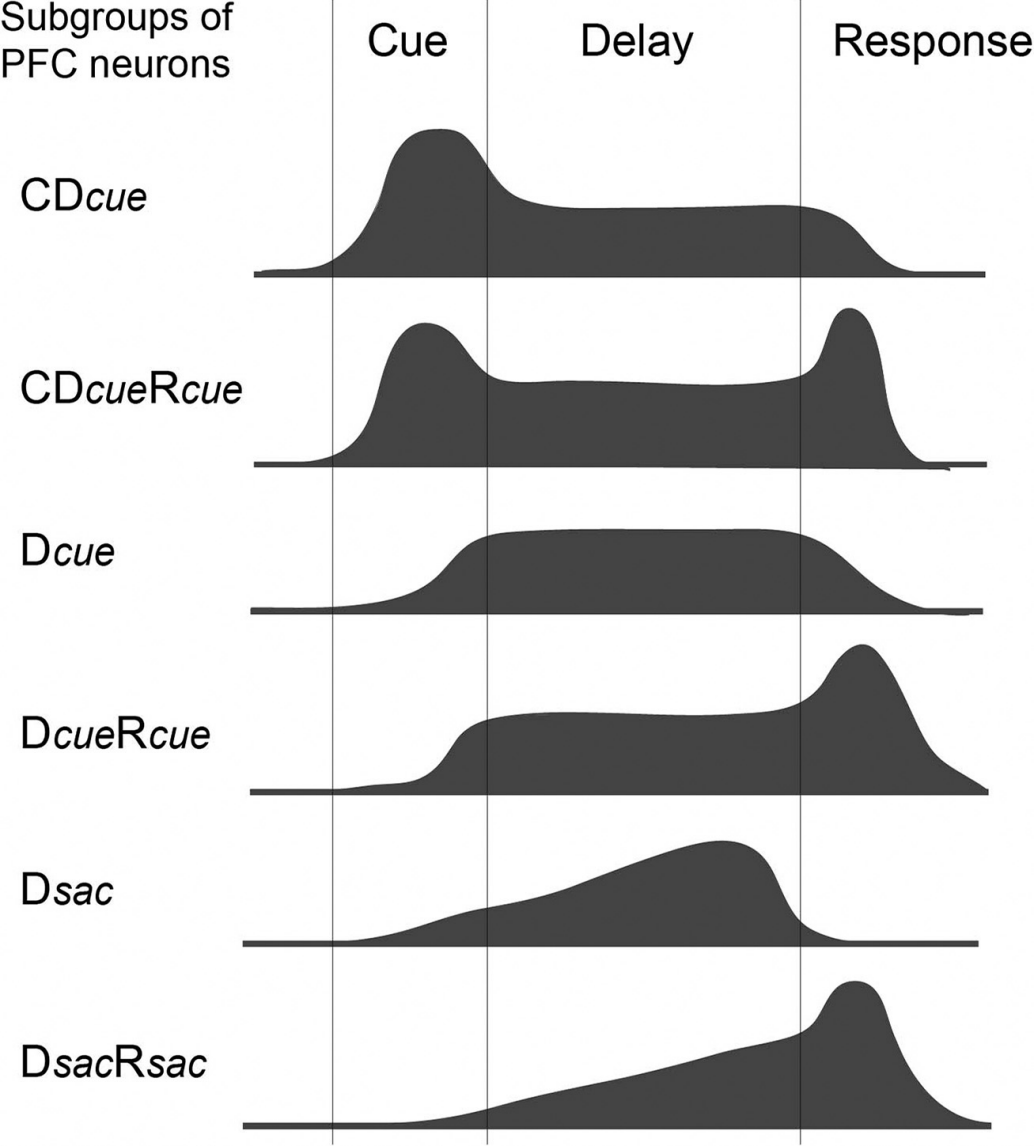
**Schematic drawings of the temporal profiles of activity for six groups (D*cue*, D*sac*, CD *cue*, D*cue*R*cue*, D*sac*R*sac*, and CD*cue*R*cue*) of DLPFC neurons (adapted from Takeda and Funahashi (2007))**.

To further understand the mechanism of information processing in the DLPFC, we estimated information flow among DLPFC neurons during spatial working memory performance. Individual DLPFC neurons exhibit one or more task-related activities. Based on the temporal pattern of neuron activity, we could determine what task-related activity each DLPFC neuron exhibited, what information (*cue direction* or *saccade direction*) each task-related activity represented, and the preferred direction of each task-related activity for each neuron. While monkeys performed ODR tasks, DLPFC neurons exhibit task-related activities, such as cue- (C), delay- (D), or response-period (R) activity, or their combinations (C&D, C&R, D&R, or C&D&R). Takeda and Funahashi (2007) classified recorded neurons into nine groups based on which task-related activity the neuron exhibited and what information (*cue direction* or *saccade direction*) each task-related activity represented (C, D*cue*, D*sac*, CD*cue*, D*cue*R*cue*, D*sac*R*sac*, D*cue*R*sac*, CD*cue*R*cue* and CD*cue*R*sac*) (Figure 3). Preferred directions were compared between task-related activities in the same DLPFC neuron or in two different neurons. In groups of neurons that exhibited CD*cue*, CD*cue*R*cue*, and CD*cue*R*sac* activities, both cue- and delay-period activities represented the direction of the visual cue, suggesting that the directional selectivity of delay-period activity is affected by the directional selectivity of cue-period activity for these neurons. In groups of neurons that exhibited D*cue*R*cue*, CD*cue*R*cue*, and D*sac*R*sac* activities, both delay- and response-period activities represented either the direction of the visual cue (D*cue*R*cue* and CD*cue*R*cue*) or the direction of the saccade (D*sac*R*sac*), suggesting that the directional selectivity of delay-period activity affects the directional selectivity of response-period activity in these neurons. The temporal profiles of delay-period activity suggest that directional cue-period activity of C, CD*cue*, and CD*cue*R*cue* groups contributes to the initiation of directional delay-period activity of CD*cue*, CD*cue*R*cue*, D*cue*, and D*cue*R*cue* groups and that directional delay-period activity of D*sac* and D*sac*R*sac* groups affects directional saccade-related activity of D*sac*R*sac*. Thus, while monkeys performed ODR tasks, information flow from neurons that exhibit directional cue-period activity to neurons that exhibit directional saccade-related activity is present in the DLPFC through neurons that exhibit directional delay-period activity. During this information flow, visual information is gradually transformed into motor information.

**Figure 4:**
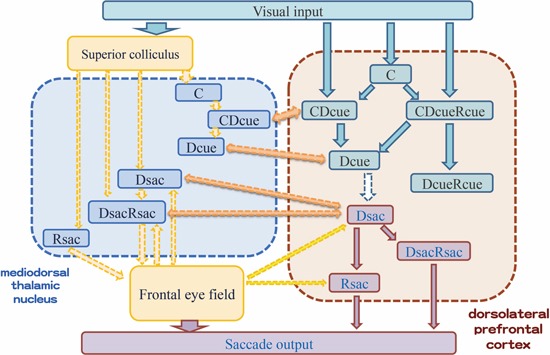
**Schematic drawing of information flow during delayed-response performance and possible interactions between DLPFC and MD neurons based on the characteristics of task-related activities of these neurons**.

As we mentioned above, all neurons with only cue-period activity represent visual information and most neurons with only response-period activity represent motor information in the DLPFC. Therefore, based on these observations, an outline of the possible information flow during spatial working memory performance in the DLPFC is shown in Figure 4. Visual inputs first activate DLPFC neurons that only have cue-period activity (C*cue*). This activation is transferred to DLPFC neurons that have both cue- and delay-period activity (C*cue*D*cue*) and then to DLPFC neurons that have only delay-period activity (D*cue*). Since all of these DLPFC neurons receive visual inputs, both cue- and delay-period activities represent visual information. However, during the delay period, prospective motor information is generated and this information is maintained in DLPFC neurons that only have delay-period activity (D*resp*). This information is transferred to DLPFC neurons with both delay- and response-period activities (D*resp*R*resp*) and then to DLPFC neurons with only response-period activity (R*resp*). A comparison of the directional selectivity of delay-period activity between the ODR and R-ODR tasks revealed that delay-period activity encoded either visual information or saccade information in the DLPFC. No delay-period activity encoded both visual and saccade information simultaneously. Therefore, prospective motor information is necessary to generate delay-period activity that encodes saccade information in the DLPFC.

In this sense, the MD can be considered a candidate of brain structures that provide information regarding prospective motor information to the DLPFC. In the MD, a majority of neurons with delay-period activity encoded saccade information (D*sac* and D*sac*R*sac*). Therefore, these MD neurons might be candidates as sources for providing prospective saccade information to the DLPFC (Figure 4).To support this idea, we need further studies to show neural interactions between the DLPFC and the MD. For example, we need to examine whether DLPFC neurons with Dsac activity have direct interactions with MD neurons with Dsac or DsacRsac activities, whether MD neurons having pre-saccadic activity provide saccade information to DLPFC neurons with Dsac, DsacRsac, or Rsac activities, or whether MD neurons having saccade-related activities are the source of post-saccadic activity observed in many DLPFC neurons. Neurophysiological studies, such as which task-related MD neurons exhibit antidromic or authodromic responses by electrical stimulations in the DLPFC, could provide important information to interpret functional interactions between the MD and the DLPFC and construct more realistic neural circuitry for these interactions than that shown in Figure 4.

Although the MD is one important brain structure for providing prospective motor information to the DLPFC, other brain structures including the FEF, the supplementary eye field, the posterior parietal cortex are also needed to be considered as strong candidates for providing prospective motor information to the DLPFC (Figure 4). We need further experiments to elucidate what information is provided from these brain structures in working memory processes.

## Involvement of other thalamic nuclei in working memory

Although the MD is one brain structure for providing prospective motor information to the DLPFC, other nuclei of the thalamus may also involve this process in working memory. For example, ventrolateral (VL) and ventroanterior (VA) thalamic neurons exhibit saccade-related activities and some of these neurons exhibited gradually increasing activities toward the initiation of the saccade (Schlag-Rey and Schlag, 1984). Tanaka (2007) also reported gradually increasing activity during the delay period of a memory-guided saccade task in the VL. Wyder et al. (2004) showed activities carrying spatial information throughout the instructed delay period of a visually guided delayed saccade task in the central thalamus. They observed two groups of delay-period activities in the central thalamus (VA and VL). One group of activity signaled the location of visible visual targets regardless of behavioral relevance, while other groups of activity signaled the locations of current goals of saccade. These activities are similar as retrospective and prospective activities observed in the DLPFC (Funahashi et al., 1993; Takeda and Funahashi, 2002) and the MD (Watanabe and Funahashi, 2004b), respectively. Recently, Kunimatsu and Tanaka (2010) examined saccade-related activities while monkeys performed either pro- or anti-saccade tasks and showed that activities of many VL and VA neurons were enhanced during the anti-saccade condition compared to the pre-saccade condition. In addition, inactivation of VL and VA nuclei by the local injection of muscimol produced an increase of error trials in the anti-saccade condition. In the anti-saccade condition, monkeys needed to maintain information regarding the location of the visual cue, but suppress an inherent response toward the visual cue. Therefore, enhanced prospective motor activity must be necessary to perform correct saccade responses by suppressing inherent reflexive responses. In human studies, Bellebaum et al. (2005) showed that patients having VL and MD lesions exhibited impairment in performing a double-step saccade task. Since two targets were presented successively in this task, the retinal direction of the second target and the saccade direction of the second saccade were different. Therefore, the subjects could not use retinal information, but needed to use corollary discharge in order to perform the second saccade correctly. Their results indicate that the VL and MD participate in the processing of corollary discharge information, as had been indicated by Sommer and Wurtz (2002, 2004).

Thus, other nuclei of the central thalamus, such as the VA and the VL, also Participate in working memory processes. The VL and the VA have been shown to project to the DLPFC (Alexander et al., 1986; Middleton and Strick, 2001; McFarland and Haber, 2002). Therefore, the VL and the VA are also possible brain structures for providing prospective motor information to the DLPFC.

## Conclusion

Working memory is a dynamic neural system that includes processes for temporarily maintaining and processing information. Working memory plays significant roles in a variety of cognitive functions, such as thinking, reasoning, decision-making, and language comprehension. Although the PFC has been known to play an important role in working memory, several lines of evidence indicate that the thalamic MD also participates in this process. Neurophysiological studies revealed that MD neurons exhibit directionally selective sustained delay-period activity while monkeys performed spatial working memory tasks. Sustained delay-period activity has been considered to be a neural correlate of the mechanism for the temporary maintenance of information. These studies also showed that most MD neurons that exhibit delay-period activity hold information regarding a motor response (prospective information), whereas a minority hold information regarding sensory cues (retrospective information). These observations suggest that the MD participates more in prospective motor aspects of working memory processes, in contrast to the PFC, which participates more in retrospective aspects such as the maintenance of sensory information. While monkeys perform spatial working memory tasks, spatial information provided by a visual cue must be transformed into motor information to perform an appropriate behavioral response. Both the MD and the PFC contain neurons that hold information regarding retrospective and prospective information, although the proportions of neurons that represent retrospective or prospective information are different between these two areas. In addition, the MD has strong reciprocal connections with the PFC. Therefore, these reciprocal connections between the MD and the PFC could play an important role in the transformation of retrospective information into prospective information in spatial working memory processes. A population analysis of neural activities revealed that the transformation of sensory-to-motor information occurred during the delay period in both the PFC and the MD. This analysis showed that population activities in the PFC hold spatial information until the late phase of the delay period and then gradually represent motor information, while population activities in the MD initially represent spatial information but then start representing motor information in the earlier phase of the delay period. These results indicate that reverberating neural circuits constructed by reciprocal connections between the MD and the PFC could be an important structure for transforming retrospective information into prospective information in spatial working memory processes.

## Conflict of interest statement

The authors declare that the research was conducted in the absence of any commercial or financial relationships that could be construed as a potential conflict of interest.
